# The effect of postoperative early lumbar drainage on delayed fever after cerebellopontine angle tumour surgery: study protocol for a randomized controlled trial

**DOI:** 10.1186/s13063-022-06950-1

**Published:** 2022-12-12

**Authors:** Yunze Zhang, Yingxi Wu, Yang Wu, Gang Zhu, Yafei Xue, Yan Qu, Tianzhi Zhao

**Affiliations:** Department of Neurosurgery, Tangdu Hospital, The Air Force Medical University, No. 569 Xinsi Road, Xi’an, Shaanxi Province 710038 People’s Republic of China

**Keywords:** Lumbar drainage, Cerebellopontine angle tumour, Randomized controlled trial, Delayed fever post-neurosurgery, Infection, Aseptic inflammation

## Abstract

**Background:**

Postoperative fever is one of the common complications in neurosurgery, and intracranial aseptic inflammation and infection are important risk factors. Continuous drainage of cerebrospinal fluid (CSF) via lumbar drainage (LD) is often used in the treatment of postoperative intracranial infection or aseptic inflammation. Compared with the previously reported placement of LD after the onset of meningitis symptoms, we designed this randomized controlled trial (RCT) to evaluate the effectiveness and safety of early drainage (1st day postoperation) of CSF using the preset lumbar cistern to prevent delayed fever (fever occurred after the third day postoperation) or reduce its treatment time after cerebellopontine angle (CPA) tumour surgery.

**Methods:**

Patients suffering from CPA tumours and who underwent resection of the tumour with an intraoperative dura opening time > 4 h are recruited for this study. The study is a 2-arm RCT to compare the early LD group and the no early LD group. Postoperative duration and rate of delayed fever and postoperative length of stay (LOS), as the main outcomes, will be compared in the two groups.

**Discussion:**

Here, we present the study design of a prospective RCT to evaluate the safety and efficacy of using preoperative preset LD to treat or reduce postoperative delayed fever.

**Trial registration:**

China Clinical Trial Registry ChiCTR2100049057. Registered on July 20, 2021.

**Table:** 

Administrative information	
Title {1}	The effect of postoperative early lumbar drainage on delayed fever after cerebellopontine angle tumour surgery: study protocol for a randomized controlled trial
Trial registration {2a and 2b}	www.chictr.org.cn identifier: ChiCTR2100049057; registered on July 20, 2021. All items can be found within the protocol
Protocol version {3}	V2.0, 2021.06.07
Funding {4}	No funding
Author details {5a}	Department of Neurosurgery, Tangdu Hospital, The Fourth Military Medical University, Xi’an, People’s Republic of China
Name and contact information for the trial sponsor {5b}	Correspondence to: Tianzhi Zhao, Department of Neurosurgery, Tangdu Hospital, The Air Force Medical University. No. 569 Xinsi Road, Xi’an, Shaanxi Province, 710038, China. Fax + 86 29 84717737. E-mail address: zhaotianzhi1981@163.com
Role of sponsor {5c}	T Z, Y Q, Y Z, and YX W originated the concept for the trial and led the development of the study design. T Z, Y Q, Y W, G Z, and Y X developed the trial standard operating procedures. Y Z, Y W, and YX W designed the data collection systems. Y Z and T Z drafted and edited the manuscript. Y Z contributed to the statistical analysis plan. All authors have read and approved the final manuscript

## Introduction

### Background and rationale {6a}

Postoperative fever is one of the common complications in neurosurgery [1, 2]. In addition to the absorption of heat and other systemic factors, such as hypostatic pneumonia, intracranial aseptic inflammation, and infection are important risk factors resulting in delayed postoperative fever [3–5]. Aseptic meningitis can be produced by bone foam, necrotic cells, haemostatic materials, repair materials, or bloody CSF [6]. It is well known that infection is caused by bacterial invasion during surgery. Fever may increase anxiety, delay recovery, significantly prolong the hospitalization days after the operation, increase medical expenses, and even increase the risk of death and disability [3, 7, 8].

In the case of intracranial infection, intravenous antibiotics alone are not sufficient. Continuous LD of CSF can remove bacteria, toxins, and necrotic substances. Therefore, it can quickly relieve the symptoms of meningeal irritation, alleviate intracranial infection, and enhance the therapeutic effect in a short time. For aseptic inflammation, such as SAH, early preventive lumbar cistern drainage can also reduce the incidence of complications, such as fever, before the appearance of fever signs [9, 10]. Therefore, we hypothesize that early LD of infectious factors or aseptic inflammatory factors play a positive role in the prevention and treatment of the two situations.

Surgery for CPA tumours located deeply and surrounding dense nerves and vasculature is very meticulous and requires a considerable amount of time, resulting in a significant increase in delayed postoperative fever. Coincidently, to reduce the traction of the cerebellum during the operation, a lumbar cistern catheter is often pre-set before the operation to release a certain amount of CSF intraoperatively [11–13]. Therefore, we design this RCT to evaluate the effectiveness and safety of early drainage of CSF using the pre-set lumbar cistern to prevent delayed fever or reduce its treatment time after CPA tumour surgery. Successful verification of this scheme may greatly optimize the perioperative treatment process of patients with complex CPA tumours and other complex intracranial tumours, providing great economic and social benefits.

### Objectives {7}

We design this randomized controlled trial (RCT) to evaluate the effectiveness and safety of early drainage (1st day postoperation) of CSF using the preset lumbar cistern to prevent delayed fever or reduce its treatment time after cerebellopontine angle (CPA) tumour surgery.

### Trial design {8}

**Fig. 1:**
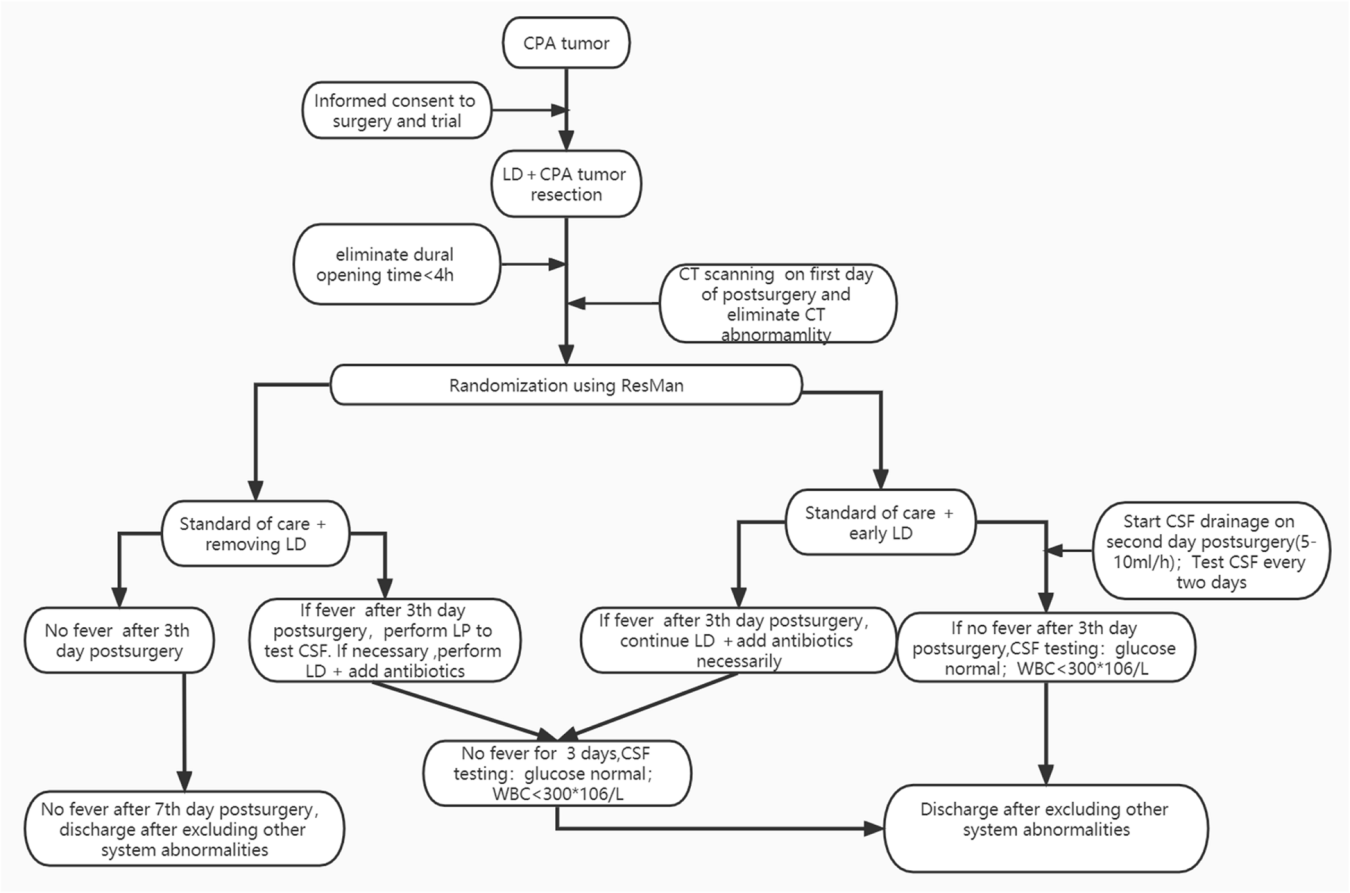
Trial flow diagram

The study is a 2-arm RCT to compare an intervention group receiving postoperative early LD and a control group receiving standard postoperative care only (Fig. 1). We hypothesize that early LD can prevent delayed fever or reduce its treatment time after CPA tumour surgery. The study is conducted by a study group treating at least 70 patients with CPA tumours per year. Data management and monitoring will be performed by Resman. Patients with CPA tumours who underwent resection of the tumour with an intraoperative dural opening time > 4 h are recruited for this study. All medical treatments are performed according to local guidelines and standard operating procedures.

## Methods: participants, interventions and outcomes

### Study setting {9}

The trial will be conducted in the Department of Neurosurgery, Tangdu Hospital, The Air Force Medical University.

### Eligibility criteria {10}

The following are the subject inclusion criteria:

- Age: 18–65 years
- First surgery for tumours in the cerebellopontine angle and dural opening time > 4 h
- Informed consent provided by the patient or his or her legal representative
- Subject exclusion criteria
- Abnormal inflammatory indicators before surgery
- Patients with severe intracranial hypertension or hydrocephalus
- Patients with severe heart liver lung and kidney insufficiency
- Patient with severe lumbar hyperostosis or intervertebral disc herniation
- Patient is the researcher or his consanguinity who may have improper informed consent
- Patients with the use of dexamethasone or other steroid medications or who have immunosuppression
- The attending doctor thinks it is inappropriate to participate in this trial
  All subjects had the right to withdraw from the trial at any time. During the trial, the researcher also has the right to terminate the trial of the subjects who meet the following conditions:
- Withdrawal from informed consent (the subjects decide to withdraw for any reason).
- The dural opening time is less than 4 h.
- The researchers believe that clinical adverse events (AEs), laboratory abnormalities, or secondary diseases (other systemic infections) indicate that it is not in the best interests of the subjects to continue to participate in the trial.
- Subject compliance is poor.
- After participating in the trial, it is found that the subjects did not meet the inclusion criteria or that the subjects are lost to follow-up.
- Subjects are imprisoned or forced to be isolated for the treatment of mental or physical diseases (such as infectious diseases).
- Subjects with allergies to the antibiotics given (meropenem and vancomycin).
- The ethics committee or regulatory body requests to terminate the trial.
- Serious violation of the protocol during the test.

Trained research staff will assess the eligibility criteria for all participants who will be performed the surgery of CPA tumour resection and ask potential participants for written informed consent for trial enrollment.

### Who will take informed consent? {26a}

Consent for study inclusion is sought after explanation and agreement to CPA tumour surgery. Thus, patients capable of consenting to CPA tumour surgery will be informed about the study details themselves and may or may not agree to participate. If a patient is incapable of consenting to the proposed treatment, the legal representative should be informed of the conditions of treatment choices and afterwards of the details of the study. A patient may be randomized if the legal representative provides informed consent to the study.

### Additional consent provisions for collection and use of participant data and biological specimens {26b}

N/A, no additional participant data and biological samples will be obtained to be stored for future studies.

### Interventions

#### Explanation for the choice of comparators {6b}

The explanation is in the “Introduction” section.

#### Intervention description {11a}

Patients with a tumour in the CPA are routinely treated with LD and tumour resection. Before opening the dura, the LD is turned on to release a certain amount of CSF slowly, and then the tumour is removed according to conventional procedures. If the dural opening time is greater than 4 h during the operation, these patients are randomly divided into the early LD group and the no early LD group. The patients’ LD is removed in the no early LD group and reserved in the early LD group.

In the no early LD group, body temperature is monitored routinely. If there is no fever after the 3rd day postoperation, the patients could be discharged, excluding other systemic abnormalities after the 6th day postoperation. If the body temperature is greater than 37.4 °C within the 3rd day postoperation, absorption heat is considered and symptomatic management will be given without any other intervention. If the body temperature is greater than 37.4 °C after the 3rd day postoperation, a lumbar puncture is performed to test the CSF. If the number of cells and white blood cells (WBCs) in CSF is increased and other systemic infections are excluded by testing the blood, urine, sputum, and stool and scanning chest CT, lumbar puncture should be performed to release CSF, or LDs should be placed to continuously drain CSF. If the body temperature is greater than 38 °C, the WBC count in the cerebrospinal fluid is increased, CSF glucose is less than 2.2 mmol/L, or CSF glucose/serum glucose is less than or equal to 0.4, vancomycin (1 g, Q12H) and meropenem (2 g, Q8H) will be administered. After treatment, if the body temperature is normal for 3 consecutive days, the routine WBC count in the cerebrospinal fluid is less than 300 × 106/L [14], the sugar is normal, the restricted antibiotics and LD can be ceased, and the discharge standard can be reached after excluding other system abnormalities. The patients will be followed up for 30 days after discharge.

In the early LD group, the LD is placed at a safe height, and the CSF drainage is controlled at approximately 150 mL/day after eliminating CT abnormalities on the first day post-surgery. Routine, biochemical, and culture tests of CSF are performed every 2 days. In addition, body temperature is routinely monitored. If the body temperature is greater than 37.4 °C within the 3rd day postoperation, absorption heat is considered and symptomatic management will be given without any other intervention. If the body temperature is normal for 72 h after the 3rd day postoperation, the routine WBC count in CSF is less than 300 × 106/L, and the CSF glucose is normal, the lumbar cistern could be removed, and the discharge standard could be reached after excluding other system abnormalities. If the body temperature is greater than 37.4 °C after the 3rd day postoperation, lumbar cistern drainage is continued. If the body temperature is greater than 38 °C, the WBC count in cerebrospinal fluid is increased, CSF glucose is less than 2.2 mmol/L, and CSF glucose/serum glucose is less than or equal to 0.4, meropenem and vancomycin are administered. After treatment, if the body temperature is normal for 3 consecutive days, the routine WBC count in CSF is less than 300 × 106/L, and the CSF glucose is normal, the restricted antibiotics and LD can be ceased and LD can be ceased, and the discharge standard can be reached after excluding other system abnormalities. The patients will be followed up for 30 days after discharge.

If the number of WBCs in CSF gradually decreases and then increases during LD drainage, the possibility of retrograde infection should be considered. In addition, LD should be removed, and lumbar puncture should be performed according to the need. If the time of LD exceeds 10 days, LD repositioning is needed in the next lumbar segment or continuous lumbar puncture is performed.

#### Criteria for discontinuing or modifying allocated interventions {11b}

When patients suffer serious complications related to LD, such as brain hernia or repeated wetness at the skin outlet of the LD tube, which increases the risk of retrograde infection, LD will be discontinued.

#### Strategies to improve adherence to interventions {11c}

Doctors, nurses, and patients’ families work together to reduce LD-related complications and improve adherence to LD.

#### Relevant concomitant care permitted or prohibited during the trial {11d}

The LD tube is placed at a safe height, and the CSF drainage is controlled at approximately 150 mL/day. Moreover, a stopcock is not allowed to open when the patient got out of bed. If a patient had a serious headache induced by intracranial hypotension, the LD catheter could temporarily be closed or raised to decrease CSF drainage. The LD catheter is replaced if it is fractured or had frequent CSF leaks at the puncture site to prevent retrograde infection of the lumbar subarachnoid. The lumbar catheter also needed to be replaced at the other lumbar space in patients who had a fever, whose lumbar catheterization lasted more than 10 days, and whose intracranial infection is not controlled completely.

Dexamethasone or other steroid medications are prohibited during the trial.

Necessary antipyretics can be permitted during the trial.

#### Provisions for post-trial care {30}

After the trial, we will provide the participant 100 yuan and exempt the cost of online diagnosis and treatment on the good doctor application for compensation.

### Outcomes {12}

The primary outcomes in the study are postoperative duration and rate of delayed fever and postoperative hospital LOS, which is designated from the date of surgery to the date of discharge. The rates of death, reoperation, and readmission caused by intracranial haemorrhage, CSF leakage, and intracranial infection at postoperative day 30 are also calculated in both groups. In addition, postoperative CSF leaks, including rhinorrhea, otorrhea, and incision leaks, are compared between the two groups according to postoperative clinical manifestations and MRI.

The other outcomes included complications (e.g. headache, nausea, vomiting, pneumocephalus, nerve root pain and numbness, brain herniation, and accidental withdrawal of LD tubes) related to LD and other systemic complications (e.g. respiratory, cardiovascular and urinary system complications, epilepsy, and venous thrombosis).

### Participant timeline {13}

**Fig. 2:**
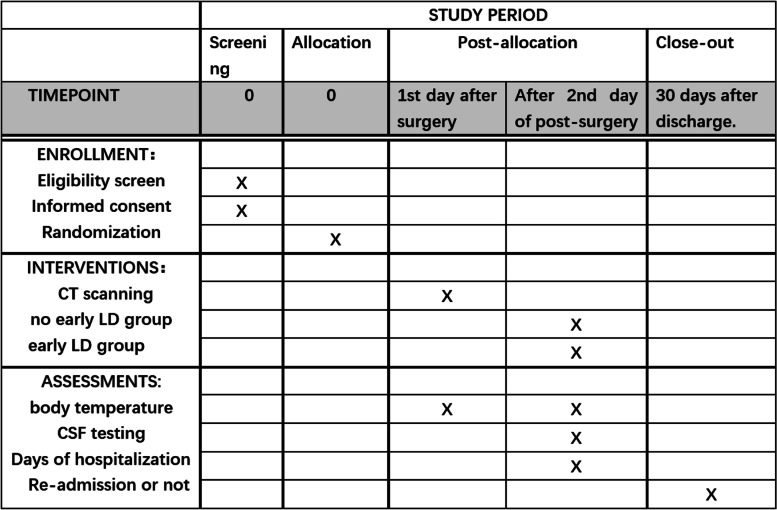
Schedule of enrolment, interventions, and assessments (SPIRIT figure)

The participant timeline is presented in Fig. 2

### Sample size {14}

To test whether the early lumbar cistern drainage group is better than the nonearly lumbar cistern drainage group. The two groups are divided into two groups at a ratio of 1:1. According to the results of an earlier review of dural opening times of greater than 4 h, the descriptive statistical result of fever time in the early lumbar cistern drainage group is 7.76 ± 3.13 days, and the descriptive statistical result of the group without early lumbar cistern drainage is 11. 23 ± 4.93 days [14]. The smaller the index value, the better the treatment effect. Setting the threshold value of superiority to 1.5 (SM value), *α* = 0.05 and *β* = 20, the sample size required for this trial is N1 = N2 = 56. If the loss of follow-up rate is 20%, 70 patients with N1 = N2 are included in each group. Therefore, 140 patients are included in the study. The sample size was calculated using SPSS (version 19.0).

### Recruitment {15}

The participant will be recruited from the inpatients of the department of neurosurgery at Tangdu Hospital, Air Force Medical University.

The Department of Neurosurgery in Tangdu Hospital is the largest neurosurgery centre in Northwest China with about 4000 intracranial tumour surgeries per year, which would provide adequate quantity of CPA tumour patients.

### Assignment of interventions: allocation

#### Sequence generation {16a}

Any patient meeting the inclusion criteria and not violating the exclusion criteria may participate in the study and be randomized to either receive a postoperative early LD or not, thus defining the two distinct groups: early LD and no early LD. Randomization is performed via a dedicated internet site accessible to the investigator (http://www.medresman.org.cn/login.aspx).

#### Concealment mechanism {16b}

The chief surgeon is unaware of the result of randomization until completing the surgery.

After surgery, there is no concealment mechanism.

#### Implementation {16c}

An independent researcher who will not be involved in patient care, outcome assessment, data collection, or data analysis generates a random number and the result of allocation (experimental group or comparison group) via the Internet site above before the initiation of the surgery. All members would be aware of the result after surgery.

### Assignment of interventions: blinding

#### Who will be blinded {17a}

After the assignment to interventions, everyone is unblinded during the study.

#### Procedure for unblinding if needed {17b}

N/A. After the assignment to interventions, everyone is unblinded during the study.

### Data collection and management

#### Plans for assessment and collection of outcomes {18a}

Work training will be held before the start of the project to provide detailed training and explanation of the trial protocol, standard operating procedures (SOPs) for study operations, and the content of case report forms (CRFs). Well-trained assessors will use CRFs to collect clinical data after participant discharging. The person in charge of CRFs checks the weekly completion and files the case report form in summary after standardization. Supervisors also should randomly check the CRFs at any time. Data will be recorded on the paper version of CRFs by designated outcome assessors and double-entered in the electronic CRFs. Any incomplete data will be recorded as unknown, missing, or not applicable.

#### Plans to promote participant retention and complete follow-up {18b}

This trial mainly involved randomized group data, baseline data, post-intervention outcome data, follow-up data, and statistical analysis result data. Randomized group data are provided and managed by the investigator responsible for the random grouping of the trial. Baseline data, post-intervention outcome data, and follow-up data are collected by dedicated data collection researchers. Statistical analysis result data are collected and provided by the researcher in charge of data statistical analysis. In order to maximize the completeness of data collection, we will enhance communication with patients. The participant who accomplishes the trial and follow-up will receive some economic compensation.

#### Data management {19}

All study-related information, such as participants’ medical records, will be kept securely in locked file cabinets with limited access. All private subject data are encrypted and protected, visible only to the project’s principal investigators, and used only for the research of the project. Principal investigators will be granted access to the cleaned dataset. To ensure confidentiality, data dispersed to project team members will be kept confidential to any identifiable participant information.

#### Confidentiality {27}

To ensure confidentiality, data dispersed to project team members will be kept confidential to any identifiable participant information.

#### Plans for collection, laboratory evaluation, and storage of biological specimens for genetic or molecular analysis in this trial/future use {33}

N/A, no additional biological specimens for genetic or molecular will be obtained in this study.

## Statistical methods

### Statistical methods for primary and secondary outcomes {20a}

All data, including patient characteristics and intraoperative and postoperative outcomes, are obtained and assessed during the hospital stay and follow-up period (30 days). Student’s *t*-test is used for continuous data with a normal distribution, and the Mann–Whitney *U*-test is employed for non-normally distributed data (e.g. duration of delayed fever). For categorical data, the chi-square test or Fisher’s exact test is performed (e.g. rate of delayed fever, extent of tumour resection, rate of postoperative complications). A significant difference was identified as a *p* value < 0.05. All data were analysed by means of SPSS (version 19.0).

### Interim analyses {21b}

Interim analyses (e.g. rate and duration of delayed fever, rate of AE and SAE) will be performed by independent statisticians and reported to the data monitoring committee (DMC).

### Methods for additional analyses (e.g. subgroup analyses) {20b}

N/A, there are no additional analyses.

### Methods in analysis to handle protocol non-adherence and any statistical methods to handle missing data {20c}

For missing data, we will use the last-observation-carried-forward method.

### Plans to give access to the full protocol, participant-level data, and statistical code {31c}

At the end of the study, a completely de-identified dataset is scheduled to be delivered to the Internet site (http://www.medresman.org.cn/login.aspx) which will be accessible to the public via a certain account.

### Oversight and monitoring

#### Composition of the coordinating centre and trial steering committee {5d}

We have developed the following committees for the trial.

Study principal investigator: Yan Qu. He proposed the research hypothesis depending on the clinical question.

Steering committee: Yan Qu (chair), Tianzhi Zhao, Yunze Zhang, Yingxi Wu, Yang Wu, Gang Zhu, and Yafei Xue. We discussed the test protocols and related materials together.

In addition, an independent data monitoring committee (DMC) will be established to monitor the trial’s quality and compliance and ensure the safety of participating patients. The DMC will be composed of three experts with expertise in neurosurgery, statistics, and trial methodology, who will examine the data every 12 months and determine if a trial should be modified or discontinued. All of them are independent of the sponsor and have no competing interests. Interim analyses will be performed by independent statisticians and reported to the DMC, which can obtain these interim results and make the final decision to terminate the trial when the rate of SAEs is greater than 2%.

#### Composition of the data monitoring committee, its role, and reporting structure {21a}

The DMC will be composed of three experts with expertise in neurosurgery, statistics, and trial methodology, who will examine the data every 12 months and determine if a trial should be modified or discontinued. All of them are independent of the sponsor and have no competing interests. Interim analyses will be performed by independent statisticians and reported to the DMC, which can obtain these interim results and make the final decision to continue, modified, or terminate the trial.

#### Adverse event reporting and harms {22}

Definition of adverse events (AEs) and severe adverse events (SAEs).

The term AEs describes any sign symptom, syndrome, or any disease (1) occurring newly in a trial participant after consent to the trial and (2) being of particular interest for the assessment of the disease or the security of the therapeutic concept. In this trial, AEs include the following:

- Complications related to lumbar drainage amount
- Complications related to the insertion of a lumbar drainage
- Any SAEs

The term AEs does not implicate a causal correlation with participation in the trial. AEs are classified into severe (SAEs) and non-severe (AEs) adverse events.

An SAE is any AEs occurring during the trial that is related to the following:

- Death
- Any life-threatening condition
- Rehospitalization or prolongation of hospitalization
- Long-term or severe restraint of the state of health

Expected and unexpected harms will be collected systematically using CTCAE v5.0, and we will report all harms.

#### Frequency and plans for auditing trial conduct {23}

The audit committee, which is independent of the sponsor and has no competing interests, will audit the trial conduct every 12 months.

#### Plans for communicating important protocol amendments to relevant parties (e.g. trial participants, ethical committees) {25}

If the protocol has changed with important amendments, we will notify ethical committees in writing and trial participants orally or by calling.

#### Dissemination plans {31a}

We will communicate the trial results to participants, healthcare professionals, the public, and other relevant groups by publishing paper.

## Discussion

Continuous drainage of CSF via LD is often used in the treatment of postoperative intracranial infection or aseptic inflammation [4, 5, 8]. Compared with the previously reported placement of LDs after the onset of meningitis symptoms, this is the first time that we propose a protocol that puts forward the concept of using early LD of CSF to treat postoperative delayed fever.

The literature reported that the infection rate after neurosurgery is 1–11%, and aseptic meningitis is more common after posterior fossa surgery with an incidence of up to 30%, leading to delayed postoperative fever [4, 15–17]. Among all the fever-related factors, the duration of the operation is one of the most important factors [18, 19]. In our previous clinical work, we found that compared with the whole operation time, the dural opening time exhibits a greater correlation with delayed fever. In our previous retrospective summary, we found that the delayed fever rate of complex intracranial tumours with a dural opening time of greater than 4 h after the operation is approximately 30%, in which aseptic inflammation accounted for approximately 20% of cases and infection accounted for approximately 10% of cases. Early lumbar drainage of cerebrospinal fluid may reduce the delayed fever rate and treatment time to a certain extent. To further evaluate the therapeutic effect of early postoperative LD on postoperative delayed fever, we designed this prospective trial for patients with CPA tumours whose dural opening time is greater than 4 h during surgery.

The use of CSF drainage via a lumbar drain during cranial surgery is a common practice for reducing ICP and enhancing exposure. Despite its common practice, the use of a lumbar drain is not without complications, including headache, nausea, and vomiting [20, 21]; abducens palsy [22]; intracranial hypotension [23]; lumbar nerve root irritation [24]; and cerebellar tonsillar herniation [25]. However, according to our experience, the related complications can be prevented and controlled through the joint efforts of doctors, patients’ families and nurses.

This protocol aims to evaluate the safety and efficacy of using preoperative pre-set LD to treat or reduce postoperative delayed fever. Moreover, in the case of encouraging results indicating that early LD could significantly reduce the duration of delayed fever postoperation and length of stay, a multicentre trial may be started.

## Trial status

The trial is currently in the recruitment phase. This study protocol is version 2 made on June 7, 2021. Recruitment commenced in August 2021 at Tangdu Hospital and is expected to be completed in August 2023.

## Data Availability

At the end of the study, a completely de-identified dataset is scheduled to be delivered to the Internet site (http://www.medresman.org.cn/login.aspx) which will be accessible to the public via a certain account.

## Abbreviations

CSF: Cerebrospinal Fluid
LD: Lumbar Drainage
RCT: Randomized Controlled Trial
CPA: Cerebellopontine Angle
SOPs: Standard Operating Procedures
CRFs: Case report Forms
AEs: Adverse Events
SAEs: Severe Adverse Events
